# Dexmedetomidine administration is associated with a reduced risk of ICU mortality in critically ill patients with ischemic stroke

**DOI:** 10.3389/fneur.2025.1571957

**Published:** 2025-08-06

**Authors:** Shuiyun Chen, Shujiang Ren, Xing Li, Kewei Liu

**Affiliations:** ^1^Department of Neurology, Nanfang Hospital, Southern Medical University, Guangzhou, China; ^2^Department of Neurology, Chongqing Medical University, Chongqing, China; ^3^Department of Interventional Medicine, Guangzhou First People’s Hospital, Second Affiliated Hospital of South China University of Technology, Guangzhou, China

**Keywords:** ischemic stroke, dexmedetomidine, intensive care unit, mortality, sedative

## Abstract

**Background:**

Although the administration of dexmedetomidine (DEX) in intensive care unit (ICU) is rapidly increasing, its potential impact on critically ill patients with ischemic stroke has not yet been explored.

**Methods:**

Patient data were extracted from the Medical Information Mart for Intensive Care IV (MIMIC-IV 3.0) database to identify patients who received DEX and those who did not. The primary outcome was ICU mortality. Statistical analyses included multivariate Cox proportional hazards regression, propensity score matching (PSM), and inverse probability of treatment weighting (IPTW) to ensure the robustness of the findings.

**Results:**

This study included 646 patients (22.8%) with ischemic stroke who received DEX treatment, and 2,182 patients who did not receive DEX in the ICU. A significant reduction in ICU mortality was observed in the DEX group compared to the non-DEX group, with an adjusted hazard ratio (HR) of 0.52 [95% confidence interval (CI) 0.40–0.68, *p* < 0.001]. Within the matched cohort, DEX administration did not show a statistically significant increased risk of bradycardia and improvement in 90-day mortality outcomes.

**Conclusion:**

These findings suggest that DEX administration may reduce ICU mortality in patients with IS.

## 1 Introduction

Stroke remains one of the leading causes of global disability and mortality (1). According to the Global Burden of Disease Study, stroke accounted for approximately 7.3 million deaths worldwide in 2021, representing 10.7% of all mortalities, of which ischemic stroke (IS) accounted for 65.3% of all cases (2). A recent multicenter retrospective study involving 952,400 patients diagnosed with acute IS found that 19.9% of these patients required ICU-level care (3). However, among stroke patients receiving ICU treatment, mortality rate have been reported to reach as high as 70% (4, 5). Therefore, it is imperative to identify effective interventions to reduce ICU mortality among critically ill patients suffering from IS.

Dexmedetomidine (DEX), a high-affinity α2-adrenergic receptor agonist, exhibits sympathetic inhibitory and anti-inflammatory properties (6–8) and is frequently used for sedation, analgesia, delirium reduction in mechanically ventilated patients in the ICU (9, 10). Recent studies have suggested that early administration of DEX is associated with reduced in-hospital mortality among patients with traumatic brain injury (11). Furthermore, DEX administration may improve outcomes in patients with acute myocardial infarction, partly due to its anti-inflammatory effects (12). However, a multicenter randomized controlled trial found that DEX administration in patients with sepsis did not improve mortality or ventilator-free days (13). At present, there is limited evidence on sedation protocols for critically ill patients, and the impact of DEX on outcomes of critically ill IS patients remain unclear. Therefore, this study aims to investigate the association between DEX administration and prognosis in patients with IS in the ICU.

## 2 Materials and methods

### 2.1 Data source

The dataset used in this study was obtained from the Medical Information Mart for Intensive Care IV (MIMIC-IV 3.0) database, a comprehensive single-center repository containing information on 364,627 critically ill patients from 2008 to 2022. This database includes detailed records on patient demographics, laboratory results, nursing progress notes, intravenous (IV) medications, fluid balance, and a wide range of other clinical variables. The database was approved for research used by the institutional review boards of the Massachusetts Institute of Technology and Beth Israel Deaconess Medical Center, with a waiver of informed consent. Additionally, one of the authors of this study obtained permission to access the dataset and extract relevant data.

### 2.2 Study design and population

**Figure 1:**
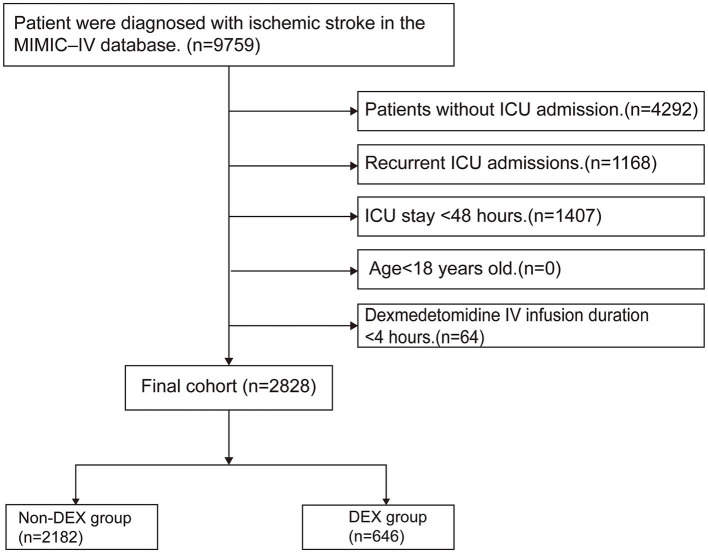
Flowchart for the inclusion of study participants. MIMIC-IV; Medical Information Mart for Intensive Care IV; ICU: intensive care unit; IV: intravenous; DEX: dexmedetomidine.

We recruited a cohort of 9,759 patients diagnosed with ischemic stroke, identified using the search term “cerebral infarction” in accordance with ICD-9 or ICD-10 coding standards. The exclusion criteria were as follows: (1) patients without ICU admission, (2) DEX IV infusion duration of less than 4 h, (3) ICU stay of less than 48 h, (4) age under 18 years, and (5) recurrent ICU admissions, with only data from the first admission being considered. A total of 2,828 patients were ultimately included in the study and divided into two groups: the DEX administration group (DEX group) and the non-DEX administration group (non-DEX group) (Figure 1).

### 2.3 Primary outcome and secondary outcomes

The primary outcome of the study was ICU mortality. Secondary outcomes included the duration of mechanical ventilation, reduction in white blood cell (WBC) count, incidence of bradycardia, length of ICU stay, and 90-day mortality. Bradycardia was defined as a heart rate below 50 beats/min following DEX administration. The reduction in WBC count was calculated as following: for the DEX group, it was determined by the difference between the initial WBC count recorded at ICU admission and the WBC count measured on the fourth day after DEX administration. For the non-DEX group, the reduction was defined as the difference between the initial WBC count at ICU admission and the WBC count obtained on the fourth day following the initial measurement.

### 2.4 Variables

Data extraction was conducted using Navicat Premium (version 16.0.6) by implementing structured query language (SQL). The extracted patient data included the following categories: (1) demographic variables: age, sex, weight and ethnicity; (2) first day vital signs: systolic blood pressure (SBP), diastolic blood pressure (DBP), mean blood pressure (MBP), respiratory rate, temperature and pulse oxygen saturation (Spo2); (3) laboratory results: WBC, red blood cell (RBC), hemoglobin, platelet, creatinine, international normalized ratio (INR), prothrombin time (PT), and partial thromboplastin time (PTT); (4) comorbidities: hypertension, diabetes, atrial fibrillation, respiratory failure, heart failure, acute myocardial infarction, sepsis, chronic renal disease stage V, liver disease and malignancy, ventilator-associated pneumonia (VAP), and delirium; (5) interventions: propofol, midazolam, antiplatelet, norepinephrine, continuous renal replacement therapy (CRRT), and mechanical ventilation; (6) clinical scores: Richmond Agitation-Sedation Scale (RASS), Glasgow coma score (GCS), Charlson comorbidity index (CCI), Sequential Organ Failure Assessment (SOFA), Acute physiology score III (APSIII), Oxford acute severity of illness score (OASIS).

### 2.5 Statistical analyses

For categorical variables, a chi-square test was conducted, with results expressed as absolute counts and proportions. For continuous data that did not follow a normal distribution, the Kruskal-Wallis test was employed, and outcomes were reported as medians interquartile ranges (IQRs).

To evaluate the relationship between DEX administration and outcomes in critically ill patients with ischemic stroke, a univariate Cox regression analysis was performed. Variables considered clinically relevant and showing a univariate association with outcomes (*p* < 0.10) were incorporated as covariates in a multivariate Cox regression model. These covariates comprised age, weight, gender, ethnicity, DBP, respiratory rate, temperature, Spo2, WBC, platelets, INR, PT, creatinine, hypertension, atrial fibrillation, chronic renal disease stage V, liver disease, malignancy, propofol, midazolam, antiplatelet, mechanical ventilation, norepinephrine, RASS, CCI, SOFA, APSIII, and OASIS.

To ensure the robustness of our findings, propensity score matching (PSM) and inverse probability of treatment weighting (IPTW) (14, 15) were employed. In the PSM model, the Cox proportional hazards regression model was used to estimate the propensity score for DEX administration. The 1:1 nearest neighbor matching method was selected, and the standardized mean difference (SMD) and *p*-value were calculated to assess the effectiveness of the PSM approach. In the IPTW model, the distribution of covariate characteristics in each group was balanced with the entire cohort by applying appropriate weighting. The baseline characteristics between the two groups were well-balanced, with SMDs for all variables being less than 0.1 (Supplementary Figure S1). Moreover, a sensitivity analysis was conducted to evaluate the robustness of the findings. In addition, a subgroup analysis was performed to investigate whether the relationship between DEX administration and ICU mortality was influenced by factors such as age, sex, specific comorbidities, the use of other sedatives, and the presence of severe disturbances of consciousness (GCS ≤ 8) upon admission.

Finally, the missing values of all variables were less than 15%, and multiple imputation, regression imputation, and mean value imputation methods were used to address the missing values. Statistical analyses were performed using Stata (version 18.0) and R Studio (version R4.4.1), with *p* < 0.05 considered statistically significant.

## 3 Results

### 3.1 Baseline characteristics of the study individuals

In our study cohort, a total of 2,828 patients admitted to the ICU with IS were enrolled. Among these, 646 patients (22.8%) received DEX following ICU admission, while 2,182 patients did not receive this treatment. Table 1 summarized the characteristics of the DEX and non-DEX groups. Compared to the non-DEX group, the DEX group was younger [65.62 (54.58–73.53) vs. 72.02 (60.92–81.68), *p* < 0.001], and had a higher proportion of male patients (62.07% vs. 48.12%; *p* < 0.001). The DEX group also exhibited higher WBC levels [10.40 (7.60–14.30) vs. 9.40 (7.00–12.30), *p* < 0.001] and a higher heart rate at admission [83.19 (73.89–96.08) vs. 79.72 (70.27–90.96), *p* < 0.001]. Furthermore, patients in the DEX group were more deeply sedated [−1.00 (−3.00–0.00) vs. 0.00 (−1.00–0.00), *p* < 0.001] and had significantly higher severity scores upon admission [e.g., SOFA: 4.00 (2.00–6.00) vs. 2.00 (1.00–4.00), *p* < 0.001]. Additionally, they were more likely to require mechanical ventilation (92.13% vs. 72.32%; *p* < 0.001) and the administration of additional sedative agents, such as propofol (96.13% vs. 72.32%; *p* < 0.001) and midazolam (42.57% vs. 17.60%; *p* < 0.001).

**Table 1:** Baseline characteristics and comparisons between the DEX group and non-DEX group before propensity score matching.

Variables	Non-DEX group (*n* = 2,182)	DEX group (*n* = 646)	*p* value	SMD
Demographics				
Age (years)	72.02 (60.92–81.68)	65.62 (54.58–73.53)	<0.001	0.430
Male, *n* (%)	1,050 (48.12%)	401 (62.07%)	<0.001	0.283
Weight (Kg)	75.30 (63.70–90.00)	80.00 (69.00–96.35)	<0.001	0.231
Ethnicity, *n* (%)			0.184	0.098
White	1,268 (58.11%)	353 (54.64%)		
Asian	73 (3.35%)	18 (2.79%)		
Black	246 (11.27%)	71 (10.99%)		
Other	595 (27.27%)	204 (31.58%)		
Vital signs in the first day				
Heart rate (bpm)	79.72 (70.27–90.96)	83.19 (73.89–96.08)	<0.001	0.248
SBP (mmHg)	130.79 (117.87–144.00)	118.83 (107.46–131.15)	<0.001	0.540
DBP (mmHg)	69.00 (59.97–79.09)	64.65 (56.70–73.18)	<0.001	0.313
MBP (mmHg)	86.55 (77.42–96.36)	80.23 (73.17–89.66)	<0.001	0.405
Respiratory rate (bpm)	18.65 (16.79–21.00)	19.50 (17.36–22.56)	<0.001	0.281
Temperature (°C)	36.89 (36.70–37.12)	36.96 (36.72–37.29)	<0.001	0.139
Spo2 (%)	97.09 (95.79–98.45)	97.67 (96.16–98.97)	<0.001	0.169
Laboratory paraments				
White blood cell, K/uL	9.40 (7.00–12.30)	10.40 (7.60–14.30)	<0.001	0.207
Red blood cell, m/uL	3.79 (3.21–4.35)	3.31 (2.83–3.91)	<0.001	0.522
Hemoglobin (g/dL)	11.30 (9.50–13.00)	9.90 (8.50–11.57)	<0.001	0.529
Platelet, K/uL	222.00 (169.00–289.00)	227.50 (155.25–302.00)	0.576	0.051
INR (ratio)	1.20 (1.10–1.36)	1.20 (1.10–1.50)	<0.001	0.156
PT (sec)	12.90 (11.70–14.90)	13.60 (12.10–16.40)	<0.001	0.156
PTT (sec)	30.90 (27.20–42.55)	33.00 (28.40–49.63)	<0.001	0.167
Creatinine, mg/dL	0.90 (0.70–1.20)	1.00 (0.70–1.60)	<0.001	0.196
Comorbidities				
Diabetes, *n* (%)	497 (22.78%)	227 (35.14%)	<0.001	0.275
Hypertension, *n* (%)	885 (40.56%)	288 (44.58%)	0.068	0.081
Atrial fibrillation, *n* (%)	889 (40.74%)	282 (43.65%)	0.187	0.059
Acute myocardial infarction, *n* (%)	128 (5.87%)	67 (10.37%)	<0.001	0.166
Respiratory failure, *n* (%)	672 (30.80%)	471 (72.91%)	<0.001	0.929
Heart failure, *n* (%)	631 (28.92%)	250 (38.70%)	<0.001	0.208
Sepsis, *n* (%)	1,063 (48.72%)	557 (86.22%)	<0.001	0.874
Chronic renal disease stage V	83 (3.80%)	52 (8.05%)	<0.001	0.181
Liver disease	32 (1.47%)	17 (2.63%)	0.046	0.082
Malignancy	448 (20.53%)	116 (17.96%)	0.150	0.065
Interventions				
Propofol, *n* (%)	875 (40.10%)	597 (92.41%)	<0.001	1.328
Midazolam, *n* (%)	384 (17.60%)	275 (42.57%)	<0.001	0.566
Antiplatelet, *n* (%)	1727 (79.15%)	517 (80.03%)	0.626	0.022
Norepinephrine, *n* (%)	327 (14.99%)	251 (38.85%)	<0.001	0.559
CRRT, *n* (%)	59 (2.70%)	80 (12.38%)	<0.001	0.373
Mechanical ventilation, *n* (%)	1,578 (72.32%)	621 (96.13%)	<0.001	0.691
Clinical several scores				
RASS	0.00 (−1.00–0.00)	−1.00 (−3.00–0.00)	<0.001	0.140
GCS	15.00 (14.00–15.00)	15.00 (14.00–15.00)	<0.001	0.150
CCI	6.00 (4.00–8.00)	5.00 (4.00–7.00)	<0.001	0.163
SOFA	2.00 (1.00–4.00)	4.00 (2.00–6.00)	<0.001	0.400
APS III	38.00 (28.00–51.00)	45.00 (34.00–62.00)	<0.001	0.406
OASIS	31.00 (26.00–37.00)	34.00 (29.00–40.00)	<0.001	0.337

DEX, dexmedetomidine; SBP, Systolic blood pressure; DBP, Diastolic blood pressure; MBP, Mean blood pressure; Spo2, Pulse oxygen saturation; INR, International normalized ratio; PT, Prothrombin time; PTT, Partial thromboplastin time; CRRT, Continuous renal replacement therapy; RASS, Richmond Agitation-Sedation Scale; GCS, Glasgow coma score; CCI, Charlson comorbidity index, SOFA: Sequential Organ Failure Assessment; APS III: Acute physiology score III; OASIS, Oxford acute severity of illness score.

### 3.2 Relationship between dexmedetomidine administration and the primary outcome

Univariate and multivariate Cox proportional hazards regression models demonstrated that the DEX group was associated with a reduced risk of ICU mortality, with an adjusted hazard ratio (HR) of 0.52 (95% confidence interval (CI) 0.40–0.68, *p* < 0.001) (Supplementary Tables S1, S2). Following PSM, 503 patients in the DEX group were matched with 503 patients in the non-DEX group using a 1:1 matching algorithm (Supplementary Table S3; Supplementary Figure S1). In the adjusted PSM model, the HR was 0.59 (95% CI: 0.44–0.81, *p* < 0.001). Furthermore, IPTW analysis further supported this association (Table 2). Details of the IPTW analyses were shown in Supplementary Table S4. Additionally, a sensitivity analysis was also conducted to examine patients with ventilator-associated pneumonia and delirium, which yielded consistent findings (Supplementary Tables S5, S6).

**Table 2:** Association between dexmedetomidine administration and ICU morality.

Models	HR	95% CI	*P* value
Unadjusted	0.52	0.41–0.67	<0.001
Multivariable adjusted	0.52	0.40–0.68	<0.001
PSM	0.59	0.44–0.81	<0.001
IPTW	0.63	0.45–0.88	0.007

HR: Hazard ratio; CI: confidence interval; PSM: propensity score matching. IPTW: inverse probability of treatment weighting.

**Figure 2:**
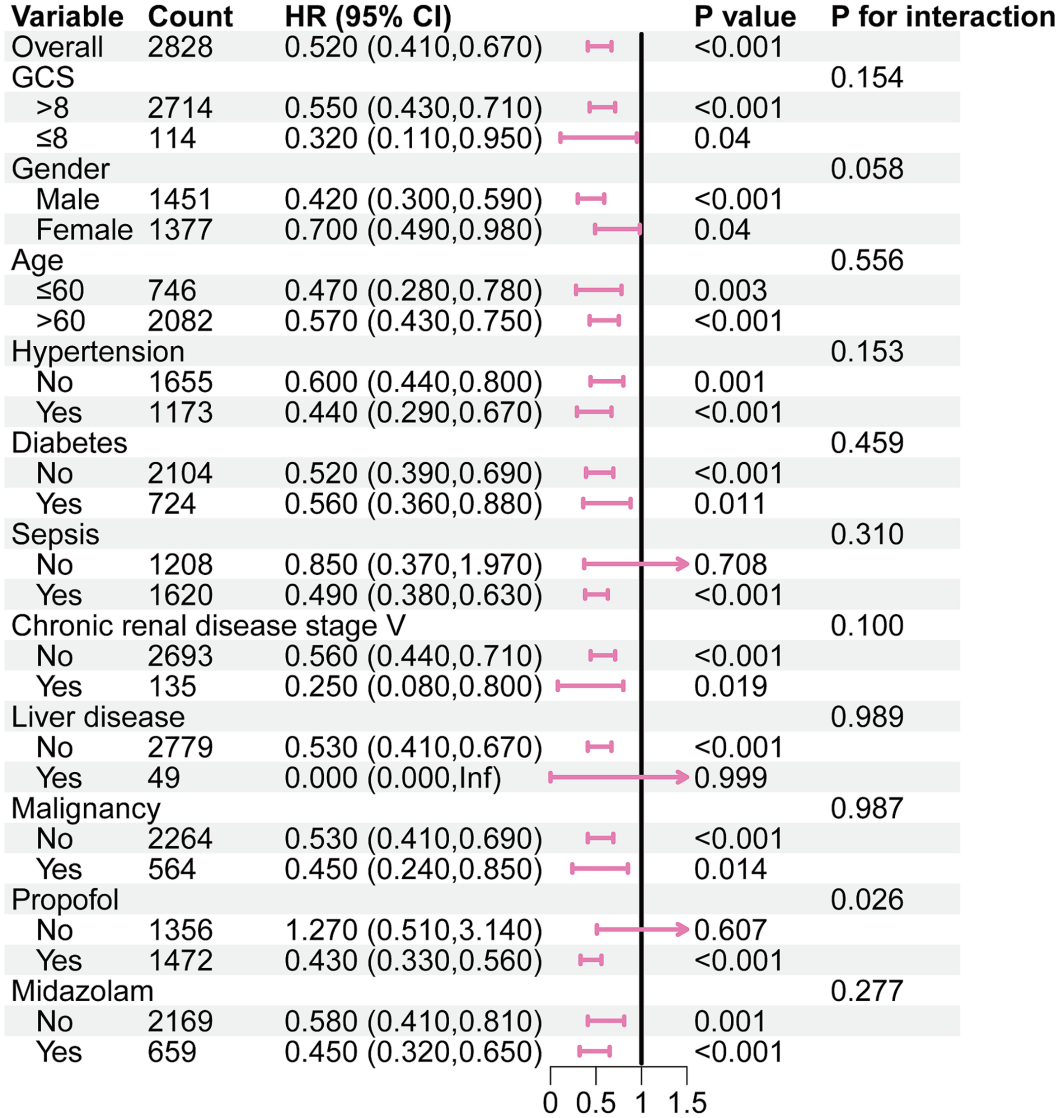
Subgroup analysis of the association between DEX administration and ICU morality in the original cohort. GCS: Glasgow coma scale.

In the subgroup analysis, the relationship between DEX administration and ICU mortality was statistically significant, regardless of whether patients were over 60 years or had severe consciousness impairment at admission, with no significant interactions observed. Notably, it seemed that the DEX administration was more prominent in patients with co-administration of propofol [0.43 (0.33–0.56) vs. 1.27 (0.51–3.14), *p* for interaction = 0.026] (Figure 2).

### 3.3 Relationship between dexmedetomidine administration and the secondary outcomes

We observed that, both in the original cohort and the matched cohort, the reduction in WBC count on the fourth day following DEX administration was significantly greater than in the group that did not receive the drug [original cohort: 1.85 (−0.90–6.00) vs. 0.30 (0.00–2.10), *p* < 0.001; matched cohort: 2.00 (−0.68–5.90) vs. 0.50 (0.00–3.00), *p* = 0.006] (Supplementary Table S7; Table 3). Although patients receiving DEX required longer mechanical ventilation [24.88 (10.00–57.00) vs. 18.12 (3.06–51.88), *p* < 0.001], there was no significant difference between the DEX and the non-DEX group in terms of the incidence of bradycardia, length of stay in the ICU, and even the 90-day mortality.

**Table 3:** Association between dexmedetomidine administration and the secondary outcomes in the matched cohort.

	Non-DEX group	DEX group	*P* value
Mechanical ventilation duration (hours)	18.12 (3.06–51.88)	24.88 (10.00–57.00)	<0.001
Reduction in WBC, K/uL	0.50 (0.00–3.00)	2.00 (−0.68–5.90)	0.006
Bradycardia, *n* (%)	256 (42.81%)	235 (39.30%)	0.217
Length of ICU stay (days)	7.79 (4.16–14.12)	8.02 (4.69–13.55)	0.396
90-day mortality, *n* (%)	206 (34.45%)	198 (33.11%)	0.625

WBC, white blood cell; ICU, intensive care unit. Bradycardia: defined as a heart rate below 50 beats/min following DEX administration.

## 4 Discussion

The results of our study demonstrated that DEX administration was associated with a reduced risk of ICU mortality in patients with IS. And we did not observe an increased risk of bradycardia associated with DEX administration.

Sedatives are essential pharmacological agents that assist clinicians in the management of critically ill patients. Currently, the sedatives employed in clinical practice encompass midazolam, propofol, and DEX, among others. A major concern related to midazolam is its elevated risk of inducing delirium (16, 17). Consequently, DEX and propofol are primarily recommended as sedatives (18). A systematic review and meta-analysis of randomized controlled trials have demonstrated that DEX may provide substantial advantages over propofol, particularly in terms of reducing the duration of mechanical ventilation and the incidence of delirium (19), as well as exhibiting more pronounced neuroprotective effects (20). At present, some studies have also demonstrated that DEX was linked to a decreased risk of new-onset atrial fibrillation in critically ill patients (21) and improved sepsis-associated acute kidney injury (22).

Our results are consistent with those of a previous multicenter, randomized controlled trial study, which indicated that early administration of DEX for sedation in mechanically ventilated ICU patients did not improve 90-day mortality (23). Similarly, another prospective multicenter study also found that early exposure to DEX was not associated with enhanced functional outcomes at 6 months in patients with traumatic brain injury (24). Nonetheless, our research provided additional evidence suggesting that the administration of DEX may significantly reduce the mortality risk within ICU in patients with ischemic stroke, thereby improving short-term adverse outcomes. In the subsequent analysis of secondary outcomes, a significant reduction in WBC count was observed in the DEX group on the fourth day post-administration, in comparison to the non-DEX group. Furthermore, another study demonstrated that DEX significantly decreased serum levels of neuron-specific enolase (NSE), S-100b, and interleukin-6 (IL-6) in patients undergoing surgery for chronic cerebral vascular stenosis (25). These findings suggested that DEX may attenuate the inflammatory response in patients with ischemic stroke, thereby potentially improving their prognosis.

DEX is increasingly being employed as a sedative for patients undergoing mechanical ventilation. In stroke patients, poststroke delirium (PSD) presented within 24 h in 25% of cases and within 72 h in nearly all cases (26). Furthermore, 10 to 15% of stroke patients necessitate mechanical ventilation (27), underscoring the critical role of sedatives in their management. The combination of DEX and propofol not only achieves optimal sedative effects but also significantly reduces the incidence of VAP in mechanically ventilated ICU patients, compared to the administration of DEX alone (28).

Several limitations should be mentioned in the present study. While the incidence of adverse reactions, such as bradycardia, was considered in this study, the evaluation of hypotension incidence remains unfeasible due to insufficient data availability. Furthermore, despite our models adjusted for RASS scores to account for sedation depth, residual confounding related to dynamic changes in sedation levels during ICU stays cannot be ruled out. Future studies should incorporate longitudinal sedation assessments to further elucidate this relationship. Additionally, although we examined the anti-inflammatory properties of DEX by observing the reduction in WBC levels, further research is necessary to comprehensively evaluate its anti-inflammatory characteristics, given the limited presence of inflammation-related indicators in the current database.

## 5 Conclusion

The administration of DEX is associated with a reduced risk of mortality in the ICU for patients suffering from IS.

## Data Availability

The datasets presented in this study can be found in online repositories. The names of the repository/repositories and accession number(s) can be found in the article/Supplementary material.
