# Supplementary material for: Dexmedetomidine administration is associated with a reduced risk of ICU mortality in critically ill patients with ischemic stroke
Source: Front Neurol. 2025 Aug 6;16:1571957. doi: 10.3389/fneur.2025.1571957 (PMC12368289; doi:10.3389/fneur.2025.1571957)
Supplement: Supplementary file 1 [file Supplementary_file_1.docx]

**Table S1** Univariate model evaluating the effects of dexmedetomidine and other important factors on ICU mortality in the original cohort.

**Table S2** Multivariate model evaluating the effects of dexmedetomidine and other important factors on ICU mortality in the original cohort.

**Table S3** Baseline characteristics and comparisons between the DEX group and non-DEX group after propensity score matching.

**Table S4** Details of the IPTW analyses on ICU mortality in the PSM cohorts.

**Table S5** Sensitivity analysis for patients with ventilator-associated pneumonia.

**Table S6** Sensitivity analysis for patients with delirium.

**Table S7** Association between dexmedetomidine administration and the secondary outcomes in the original cohort.

**Figure S1** Standard mean differences in covariates between the DEX group and the non-DEX group for the original cohort and the matched cohort.

**Table S1** Univariate model evaluating the effects of dexmedetomidine and other important factors on ICU mortality in the original cohort.

| Variables | HR (95% CI) | P value |
| --- | --- | --- |
| Dexmedetomidine | 0.52 (0.41-0.67) | <0.001 |
| Demographics |  |  |
| Age | 1.02 (1.02-1.03) | <0.001 |
| Male | 1.22 (1.00-1.50) | 0.054 |
| Weight | 1.00 (0.99-1.00) | 0.042 |
| Ethnicity |  |  |
| White | 0.72 (0.58-0.90) | 0.004 |
| Asian | 0.89 (0.49-1.61) | 0.7 |
| Black | 0.76 (0.54-1.07) | 0.12 |
| Other | Reference |  |
| Vital signs in the first day |  |  |
| Heart rate | 1.00 (1.00-1.01) | 0.273 |
| SBP | 1.00 (1.00-1.01) | 0.669 |
| DBP | 0.99 (0.98-1.00) | 0.023 |
| MBP | 0.99 (0.99-1.00) | 0.224 |
| Respiratory rate | 1.03 (1.01-1.06) | 0.006 |
| Temperature | 0.84 (0.71-0.99) | 0.042 |
| Spo2 | 1.06 (1.01-1.12) | 0.022 |
| Laboratory paraments |  |  |
| White blood cell | 1.03 (1.02-1.04) | <0.001 |
| Red blood cell | 1.02 (0.89-1.17) | 0.789 |
| Hemoglobin | 1.01 (0.96-1.06) | 0.681 |
| Platelet | 1.00 (1.00-1.00) | <0.001 |
| INR | 1.15 (0.99-1.33) | 0.059 |
| PT | 1.01 (1.00-1.03) | 0.046 |
| PTT | 1.00 (1.00-1.01) | 0.125 |
| Creatinine | 1.06 (1.01-1.11) | 0.015 |
| Comorbidities |  |  |
| Diabetes | 0.84 (0.66-1.06) | 0.146 |
| Hypertension | 0.78 (0.62-0.96) | 0.022 |
| Atrial fibrillation | 1.20 (0.98-1.48) | 0.082 |
| Acute myocardial infarction | 1.18 (0.82-1.71) | 0.375 |
| Respiratory failure | 1.07 (0.86-1.34) | 0.553 |
| Heart failure | 1.09 (0.87-0.35) | 0.459 |
| Sepsis | 0.97 (0.74-1.27) | 0.809 |
| Chronic renal disease stage V | 0.67 (0.41-1.10) | 0.113 |
| Liver disease | 0.45 (0.17-1.22) | 0.116 |
| Malignancy | 1.01 (0.77-1.33) | 0.941 |
| Interventions |  |  |
| Propofol | 1.29 (0.99-1.66) | 0.056 |
| Midazolam | 0.97 (0.78-1.20) | 0.755 |

**Table S1 (continued)**

| Variables | HR (95% CI) | P value |
| --- | --- | --- |
| Antiplatelet | 0.47 (0.37-0.58) | <0.001 |
| Norepinephrine | 1.48 (1.20-1.84) | <0.001 |
| CRRT | 1.21 (0.90-1.63) | 0.216 |
| Mechanical ventilation | 7.54 (3.10-18.30) | <0.001 |
| Clinical several scores |  |  |
| RASS | 0.67 (0.64-0.71) | <0.001 |
| GCS | 0.97 (0.93-1.01) | 0.122 |
| CCI | 1.10 (1.07-1.14) | <0.001 |
| SOFA | 1.13 (1.10-1.16) | <0.001 |
| APS III | 1.01 (1.01-1.02) | <0.001 |
| OASIS | 1.05 (1.04-1.06) | <0.001 |

SBP, Systolic blood pressure; DBP, Diastolic blood pressure; MBP, Mean blood pressure; Spo2, Pulse oxygen saturation; INR, International normalized ratio; PT, Prothrombin time; PTT, Partial thromboplastin time; CRRT, Continuous renal replacement therapy; RASS, Richmond Agitation-Sedation Scale; GCS, Glasgow coma score; CCI, Charlson comorbidity index, SOFA: Sequential Organ Failure Assessment; APS III: Acute physiology score III; OASIS, Oxford acute severity of illness score.

**Table S2** Multivariate model evaluating the effects of dexmedetomidine and other important factors on ICU mortality in the original cohort.

| Variables | HR (95% CI) | P value |
| --- | --- | --- |
| Dexmedetomidine | 0.52 (0.40-0.68) | <0.001 |
| Demographics |  |  |
| Age | 1.02 (1.01-1.03) | <0.001 |
| Male | 1.03 (0.82-1.29) | 0.816 |
| Weight | 1.00 (0.99-1.00) | 0.636 |
| Ethnicity |  |  |
| White | 0.89 (0.70-1.13) | 0.334 |
| Asian | 0.83 (0.45-1.55) | 0.566 |
| Black | 0.75 (0.52-1.09) | 0.130 |
| Other | Reference |  |
| Vital signs in the first day | |  |
| DBP | 1.01 (1.00-1.02) | 0.215 |
| Respiratory rate | 1.01 (0.98-1.04) | 0.474 |
| Temperature | 0.93 (0.79-1.10) | 0.405 |
| Spo2 | 1.05 (1.00-1.11) | 0.107 |
| Laboratory paraments | |  |
| White blood cell | 1.01 (0.99-1.02) | 0.332 |
| Platelet | 1.00 (1.00-1.00) | 0.033 |
| INR | 0.38 (0.05-2.98) | 0.354 |
| PT | 1.12 (0.92-1.35) | 0.270 |
| Creatinine | 1.00 (0.91-1.09) | 0.974 |
| Comorbidities |  |  |
| Hypertension | 0.97 (0.76-1.23) | 0.780 |
| Atrial fibrillation | 1.07 (0.84-1.35) | 0.587 |
| Chronic renal disease stage V | 0.91 (0.53-1.56) | 0.731 |
| Liver disease | 0.31 (0.11-0.91) | 0.032 |
| Malignancy | 1.05 (0.78-1.41) | 0.742 |
| Interventions |  |  |
| Propofol | 0.84 (0.62-1.12) | 0.240 |
| Antiplatelet | 0.66 (0.52-0.84) | <0.001 |
| Norepinephrine | 1.15 (0.90-1.48) | 0.264 |
| Mechanical ventilation | 3.95 (1.59-9.83) | 0.003 |
| Clinical several scores | |  |
| RASS | 0.73 (0.68-0.78) | <0.001 |
| CCI | 1.04 (0.99-1.09) | 0.144 |
| SOFA | 1.07 (1.03-1.11) | <0.001 |
| APS III | 1.00 (0.99-1.01) | 0.769 |
| OASIS | 1.02 (1.00-1.04) | 0.022 |

DBP, Diastolic blood pressure; Spo2, Pulse oxygen saturation; INR, International normalized ratio; PT, Prothrombin time; RASS, Richmond Agitation-Sedation Scale; CCI, Charlson comorbidity index, SOFA: Sequential Organ Failure Assessment; APS III: Acute physiology score III; OASIS, Oxford acute severity of illness score.

**Table S3** Baseline characteristics and comparisons between the DEX group and non-DEX group after propensity score matching.

| Vables | Non-DEX group (n=503) | DEX group  (n=503) | *P* value | SMD |
| --- | --- | --- | --- | --- |
| Demographics |  |  |  |  |
| Age (years) | 66.77 (56.41-76.93) | 67.54 (56.14-75.69) | 0.815 | 0.017 |
| Male, n (%) | 305 (60.64%) | 296 (58.85%) | 0.563 | 0.036 |
| Weight (Kg) | 77.90 (67.00-92.55) | 78.40 (68.35-94.00) | 0.406 | 0.009 |
| Ethnicity, n (%) |  |  | 0.526 | 0.040 |
| White | 285 (56.66%) | 275 (54.67%) |  |  |
| Asian | 12 (2.39%) | 14 (2.78%) |  |  |
| Black | 58 (11.53%) | 58 (11.53%) |  |  |
| Other | 148 (29.42%) | 156 (31.01%) |  |  |
| Vital signs in the first day |  |  |  |  |
| Heart rate (bpm) | 83.52 (72.99-94.38) | 82.82 (73.63-95.46) | 0.878 | 0.003 |
| SBP (mmHg) | 121.67 (109.77-133.48) | 120.96 (109.11-133.71) | 0.959 | 0.017 |
| DBP (mmHg) | 65.04 (56.64-75.04) | 64.94 (56.42-73.58) | 0.783 | 0.001 |
| MBP (mmHg) | 81.54 (73.52-90.28) | 80.96 (73.38-89.92) | 0.867 | 0.005 |
| Respiratory rate (bpm) | 19.19 (17.03-22.35) | 19.30 (17.12-22.20) | 0.768 | 0.005 |
| Temperature (°C) | 36.93 (36.67-37.29) | 36.96 (36.72-37.29) | 0.372 | 0.022 |
| Spo2 (%) | 97.76 (96.27-99.09) | 97.76 (96.23-99.00) | 0.863 | 0.013 |
| Laboratory paraments |  |  |  |  |
| White blood cell, K/uL | 10.30 (7.45-13.85) | 10.20 (7.35-14.00) | 0.961 | 0.009 |
| Red blood cell, m/uL | 3.34 (2.87-3.99) | 3.40 (2.91-4.00) | 0.492 | 0.037 |
| Hemoglobin (g/dL) | 10.10 (8.60-11.70) | 10.10 (8.70-11.70) | 0.923 | 0.007 |
| Platelet, K/uL | 218.00 (154.50-298.00) | 230.00 (162.50-304.00) | 0.210 | 0.049 |
| INR (ratio) | 1.20 (1.10-1.40) | 1.20 (1.10-1.50) | 0.529 | 0.018 |
| PT (sec) | 13.40 (12.10-15.70) | 13.50 (12.10-16.10) | 0.866 | 0.019 |
| PTT (sec) | 32.00 (27.60-46.75) | 32.00 (28.10-47.85) | 0.514 | 0.008 |
| Creatinine, mg/dL | 1.00 (0.70-1.50) | 1.00 (0.70-1.50) | 0.965 | 0.038 |
| Comorbidities |  |  |  |  |
| Diabetes, n (%) | 141 (28.03%) | 142 (28.23%) | 0.944 | 0.004 |
| Hypertension, n (%) | 199 (39.56%) | 200 (39.76%) | 0.949 | 0.004 |
| Atrial fibrillation, n (%) | 214 (42.54%) | 221 (43.94%) | 0.656 | 0.028 |

**Table S3 (continued)**

| Vables | Non-DEX group (n=497) | DEX group (n=497) | *P* value | SMD |
| --- | --- | --- | --- | --- |
| Acute myocardial infarction, n (%) | 50 (9.94%) | 52 (10.34%) | 0.835 | 0.013 |
| Respiratory failure, n (%) | 343 (68.19%) | 338 (67.20%) | 0.736 | 0.021 |
| Heart failure, n (%) | 185 (36.78%) | 187 (37.18%) | 0.896 | 0.008 |
| Sepsis, n (%) | 415 (82.50%) | 417 (82.90%) | 0.868 | 0.011 |
| Chronic renal disease stage V | 38 (7.55%) | 39 (7.75%) | 0.906 | 0.007 |
| Liver disease | 13 (2.58%) | 14 (2.78%) | 0.845 | 0.012 |
| Malignancy | 101 (20.08%) | 98 (19.48%) | 0.812 | 0.015 |
| Interventions |  |  |  |  |
| Propofol, n (%) | 451 (89.66%) | 454 (90.26%) | 0.753 | 0.020 |
| Midazolam, n (%) | 199 (39.56%) | 196 (38.97%) | 0.846 | 0.012 |
| Antiplatelet, n (%) | 394 (78.33%) | 400 (79.52%) | 0.643 | 0.029 |
| Norepinephrine, n (%) | 176 (34.99%) | 174 (34.59%) | 0.895 | 0.008 |
| CRRT, n (%) | 40 (7.95%) | 42 (8.35%) | 0.818 | 0.015 |
| Mechanical ventilation, n (%) | 477 (94.83%) | 479 (95.23%) | 0.772 | 0.018 |
| Clinical several scores |  |  |  |  |
| RASS | -1.00 (-3.00-0.00) | -1.00 (-3.00-0.00) | 0.648 | 0.050 |
| GCS | 15.00 (14.00-15.00) | 15.00 (14.00-15.00) | 0.235 | 0.016 |
| CCI | 6.00 (4.00-8.00) | 5.00 (4.00-7.00) | 0.495 | 0.036 |
| SOFA | 3.00 (2.00-5.00) | 4.00 (2.00-6.00) | 0.406 | 0.002 |
| APS III | 45.00 (31.00-61.00) | 44.00 (34.00-59.50) | 0.700 | 0.004 |
| OASIS | 34.00 (28.50-41.00) | 34.00 (29.00-40.00) | 0.862 | 0.012 |

SBP, Systolic blood pressure; DBP, Diastolic blood pressure; MBP, Mean blood pressure; Spo2, Pulse oxygen saturation; INR, International normalized ratio; PT, Prothrombin time; PTT, Partial thromboplastin time; CRRT, Continuous renal replacement therapy; RASS, Richmond Agitation-Sedation Scale; GCS, Glasgow coma score; CCI, Charlson comorbidity index, SOFA: Sequential Organ Failure Assessment; APS III: Acute physiology score III; OASIS, Oxford acute severity of illness score.

**Table S4** Details of the IPTW analyses on ICU mortality in the PSM cohorts.

| Variables | HR (95% CI) | *P* value |
| --- | --- | --- |
| Dexmedetomidine | 0.63 (0.45-0.88) | 0.007 |
| Demographics |  |  |
| Age | 1.01 (1.00-1.03) | 0.149 |
| Male | 1.05 (0.80-1.38) | 0.702 |
| Weight | 1.00 (0.99-1.00) | 0.268 |
| Ethnicity |  |  |
| White | 0.87 (0.62-1.23) | 0.429 |
| Asian | 0.60 (0.28-1.27) | 0.179 |
| Black | 1.03 (0.61-1.73) | 0.922 |
| Other | Reference |  |
| Vital signs in the first day | |  |
| DBP | 1.01 (1.00-1.02) | 0.141 |
| Respiratory rate | 1.00 (0.95-1.03) | 0.554 |
| Temperature | 0.86 (0.77-1.12) | 0.434 |
| Spo2 | 1.10 (0.98-1.15) | 0.165 |
| Laboratory paraments | |  |
| White blood cell | 1.00 (0.97-1.03) | 0.963 |
| Platelet | 1.00 (1.00-1.00) | 0.117 |
| INR | 0.40(0.04-4.55) | 0.461 |
| PT | 1.09 (0.87-1.37) | 0.469 |
| Creatinine | 0.98 (0.89-1.09) | 0.758 |
| Comorbidities | |  |
| Hypertension | 0.93 (0.66-1.31) | 0.668 |
| Atrial fibrillation | 1.10 (0.76-1.60) | 0.611 |
| Chronic renal disease stage V | 0.68 (0.38-1.23) | 0.201 |
| Liver disease | 0.25 (0.05-1.17) | 0.079 |
| Malignancy | 0.87 (0.57-1.31) | 0.500 |
| Interventions | |  |
| Propofol | 0.67 (0.40-1.12) | 0.126 |
| Antiplatelet | 0.68 (0.47-0.97) | 0.035 |
| Norepinephrine | 1.15 (0.74-1.78) | 0.539 |
| Mechanical ventilation | 5.97 (2.15-16.55) | <0.001 |
| Clinical several scores | |  |
| RASS | 0.73 (0.67-0.80) | <0.001 |
| CCI | 1.11 (1.02-1.21) | 0.021 |
| SOFA | 1.09 (1.03-1.15) | 0.004 |
| APS III | 1.00 (0.99-1.01) | 0.761 |
| OASIS | 1.02 (1.00-1.04) | 0.038 |

IPTW, inverse probability of treatment weighting; PSM, propensity score matching; DBP, Diastolic blood pressure; Spo2, Pulse oxygen saturation; INR, International normalized ratio; PT, Prothrombin time; RASS, Richmond Agitation-Sedation Scale; CCI, Charlson comorbidity index, SOFA: Sequential Organ Failure Assessment; APS III: Acute physiology score III; OASIS, Oxford acute severity of illness score.

**Table S5** Sensitivity analysis for patients with ventilator-associated pneumonia.

| Models | HR | 95% CI | *P* Value |
| --- | --- | --- | --- |
| Multivariable adjusted | 0.57 | 0.44-0.75 | <0.001 |
| PSM | 0.61 | 0.44-0.84 | 0.003 |
| IPSW | 0.58 | 0.42-0.80 | <0.001 |

HR: Hazard ratio; CI: confidence interval; PSM: propensity score matching. IPTW: inverse probability of treatment weighting.

**Table S6** Sensitivity analysis for patients with delirium.

| Models | HR | 95% CI | *P* Value |
| --- | --- | --- | --- |
| Multivariable adjusted | 0.55 | 0.42-0.72 | <0.001 |
| PSM | 0.54 | 0.39-0.74 | <0.001 |
| IPSW | 0.48 | 0.35-0.66 | <0.001 |

HR: Hazard ratio; CI: confidence interval; PSM: propensity score matching. IPTW: inverse probability of treatment weighting.

**Table S7** Association between dexmedetomidine administration and the secondary outcomes in the original cohort.

|  | Non-DEX group | DEX group | P value |
| --- | --- | --- | --- |
| Mechanical ventilation duration (hours) | 12.00 (0.00-42.00) | 24.00 (10.00-56.72) | <0.001 |
| Reduction in WBC, K/uL | 0.30 (0.00-2.10) | 1.85 (-0.90-6.00) | <0.001 |
| Bradycardia, n (%) | 619 (28.37%) | 264 (40.87%) | <0.001 |
| Length of ICU stay (days) | 4.65 (2.97-7.80) | 8.57 (4.84-15.85) | <0.001 |
| 90-day mortality, n (%) | 703 (32.22%) | 211 (32.66%) | 0.832 |

WBC, white blood cell; ICU, intensive care unit. Bradycardia: defined as a heart rate below 50 beats/min following DEX administration.


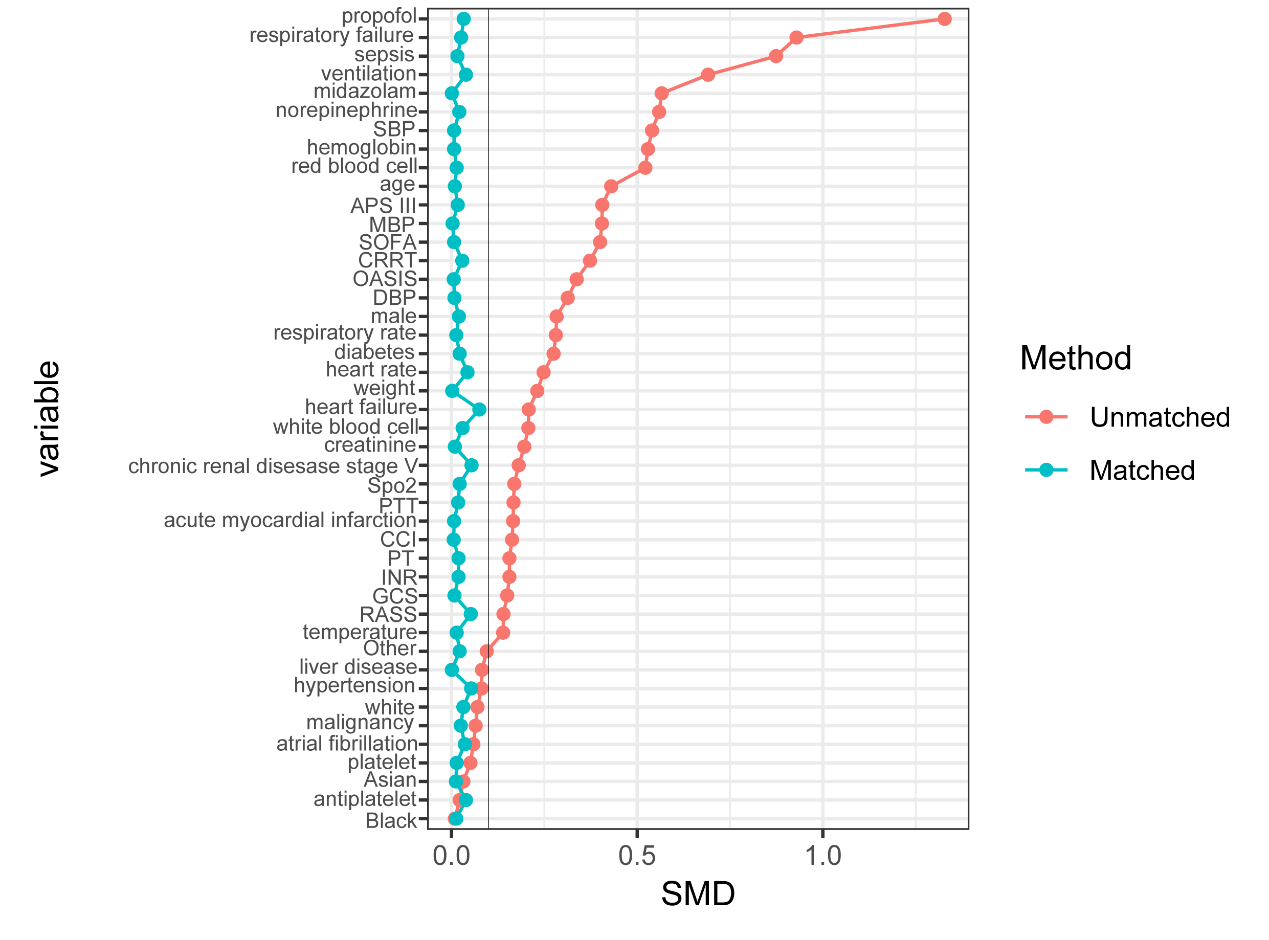


**Figure S1** Standard mean differences in covariates between the DEX group and the non-DEX group for the original cohort and the matched cohort.

SBP, Systolic blood pressure; DBP, Diastolic blood pressure; MBP, Mean blood pressure; Spo2, Pulse oxygen saturation; INR, International normalized ratio; PT, Prothrombin time; PTT, Partial thromboplastin time; CRRT, Continuous renal replacement therapy; RASS, Richmond Agitation-Sedation Scale; GCS, Glasgow coma score; CCI, Charlson comorbidity index, SOFA: Sequential Organ Failure Assessment; APS III: Acute physiology score III; OASIS, Oxford acute severity of illness score.
